# An Unusual Association: Silver-Russell Syndrome and Ectopic Thyroid

**DOI:** 10.7759/cureus.24837

**Published:** 2022-05-09

**Authors:** Fatima-Zahra Lahmamssi, Loubna Saadaoui, Hayat Aynaou, Houda Salhi, Hanan El Ouahabi

**Affiliations:** 1 Department of Endocrinology, Diabetology, Metabolic Diseases and Nutrition, Hassan II University Hospital, Fes, MAR; 2 Laboratory of Epidemiology and Research in Health Sciences, Faculty of Medicine and Pharmacy, Sidi Mohamed Ben Abdellah University, FES, MAR

**Keywords:** silver-russell syndrome, stature-ponderal delay, thyroid dysgenesis, dysmorphia, asymmetry

## Abstract

Silver-Russell syndrome (SRS) is a rare genetic disorder that combines intrauterine growth retardation, facial dysmorphia, and limb asymmetry. We report the case of a patient diagnosed with SRS on a cluster of clinical arguments, associated with thyroid dysgenesis. We report the case of a 16-year-old patient diagnosed with SRS based on the following clinical findings: hypotrophy at birth, severe stature-ponderal delay (-4DS), hemihypertrophy of the body, macrocephaly, and prominent forehead with severe psychomotor and intellectual delay (IQ < 70). The Netchine-Harbison score is rated at 6/6, hence the performance of a molecular study, the results of which are in progress. Biological and radiological exploration has objectified deep hypothyroidism on a sublingual thyroid for which he was treated with a hormone replacement therapy with L-thyroxine. This association has never been reported in the literature. We report through this case the interest in a morphological assessment in search of other anomalies, which can be associated to improve the management of SRS.

## Introduction

Silver-Russell syndrome (SRS) is a rare genetic disorder with an estimated prevalence of 1/100,000 [1,2]. It combines severe intrauterine growth retardation, postnatal weight-bearing delay, craniofacial dysmorphia, and limb asymmetry grouped in the Netchine-Harbison score [1]. Its etiology is an anomaly of parental imprinting [3]. Its management is based on symptomatic treatment, growth hormone use, and/or bone lengthening surgery in the hope of relieving the psychological suffering of patients [3]. We report the observation of a patient, in whom this syndrome is associated with thyroid ectopy, not reported before in the literature.

## Case presentation

**Figure 1:**
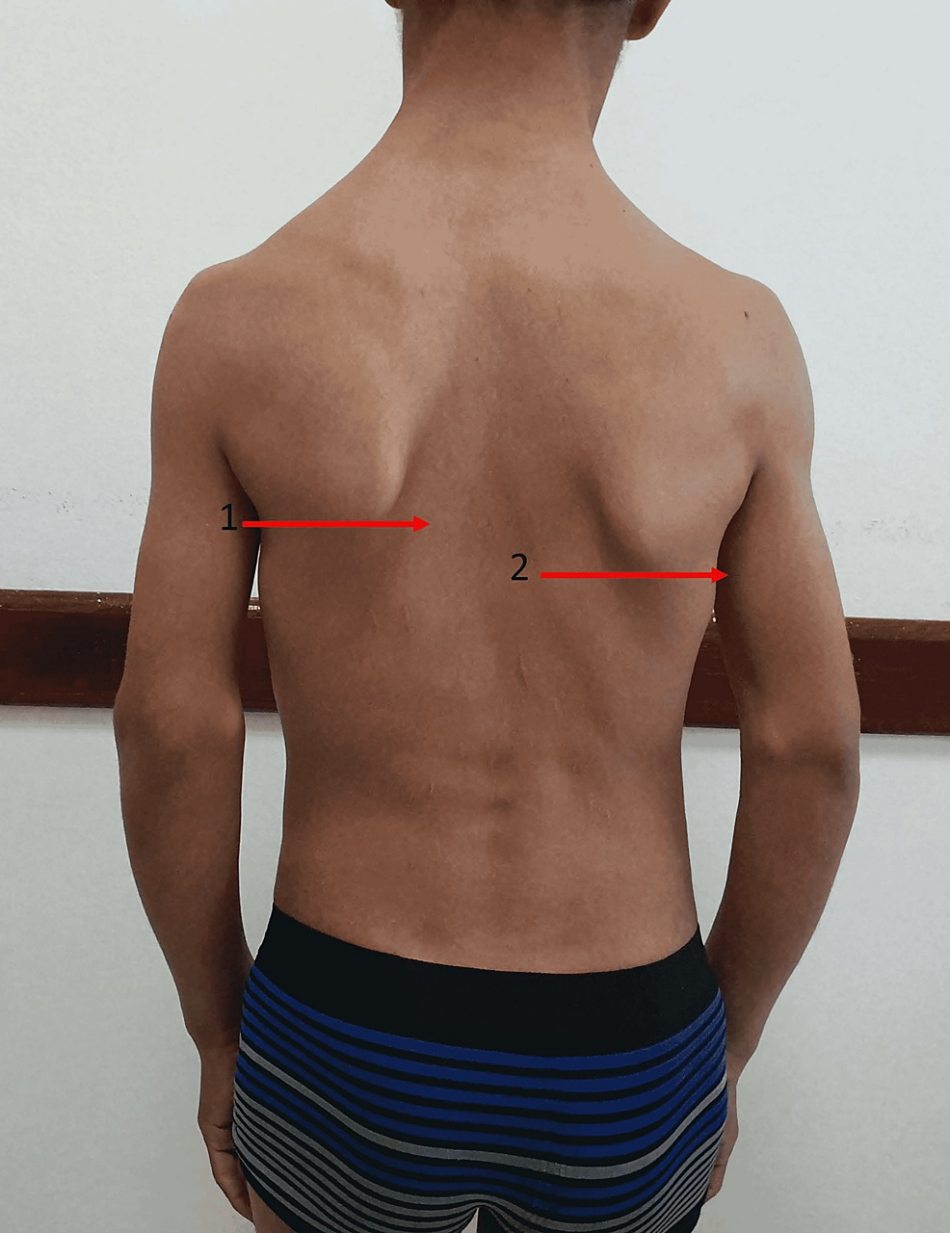
Trunk asymmetry Our patient presents body asymmetry with a very marked scapular asymmetry in this image. The difference between the two scapular regions is 1 cm. Arrow 1: lower limit of the left scapula Arrow 2: lower limit of the right scapula

**Figure 2:**
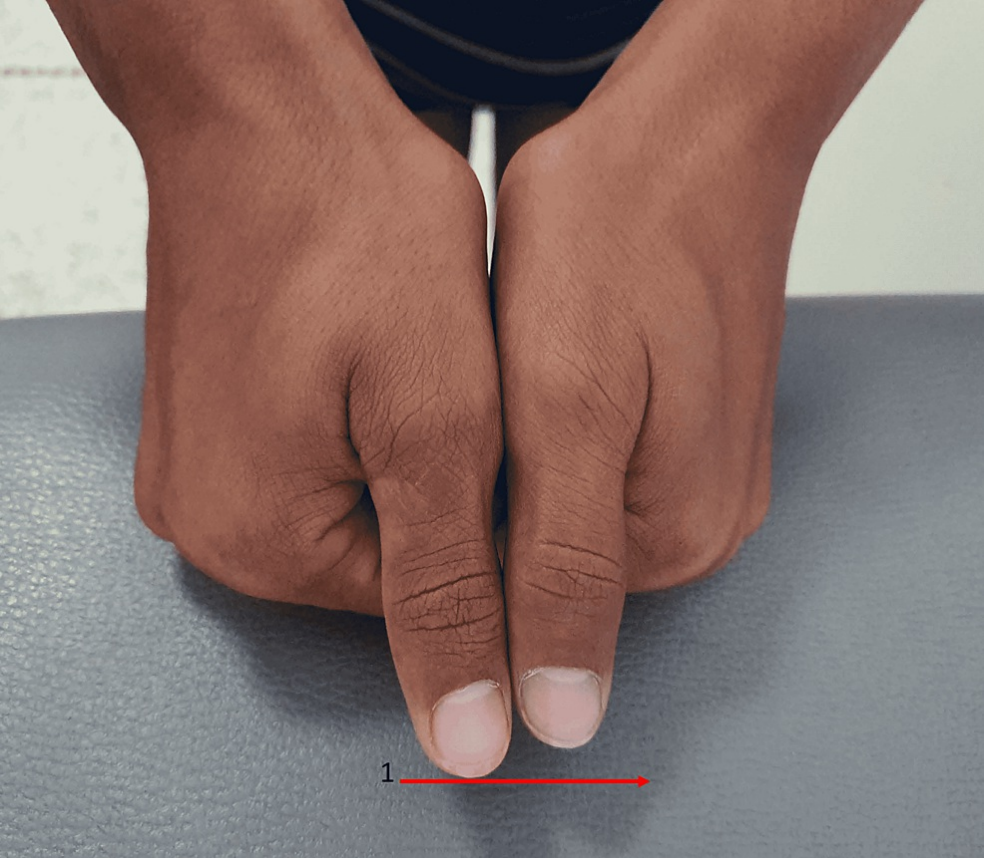
Asymmetry of the upper limbs Our patient presents asymmetry of the upper limbs with the right upper limb longer than the left upper limb. Arrow 1: asymmetry between the two hands

We report the case of a 16-year-old patient from a non-consanguineous marriage. He was admitted to the hospital for investigation of a stature-ponderal ​​delay. The interrogation revealed intrauterine growth retardation with low birth weight and profound mental retardation (IQ < 70) evolving since birth. The patient also has a delay in psychomotor development, walking, and closure of the anterior fontanel without eating difficulties or hypoglycemia. There is no similar case in the family, and there is no family history of thyroid ectopy or hypothyroidism. The clinical examination revealed a stature-ponderal delay: weight, -2.8DS; height, -4DS; and BMI, 14.8 kg/m^2^ (underweight according to the International Obesity Taskforce (IOTF) curve) in a patient at the beginning of puberty. The patient presented a dysmorphic syndrome made of a triangular face: a prominent forehead, cranial perimeter at +1.8DS, and ogival palate without dental anomalies. He has body asymmetry, as can be seen in Figure 1 (trunk asymmetry) and Figure 2 (limb asymmetry)

**Figure 3:**
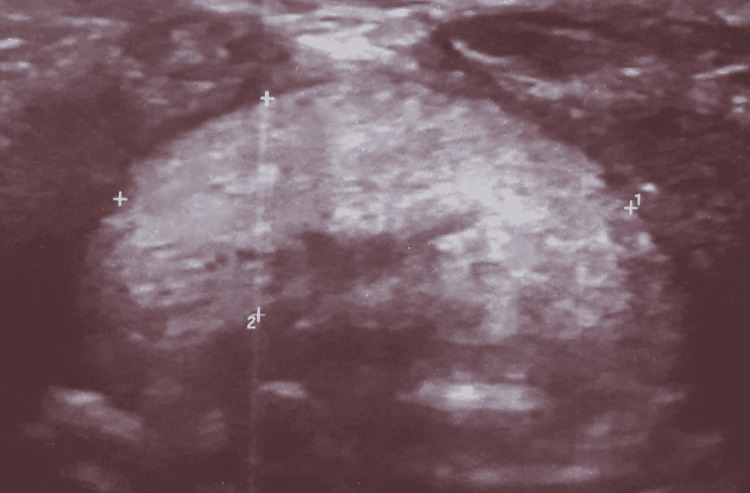
Ultrasound image showing the ectopic thyroid Ultrasound image in a transect through the upper limit of the hyoid bone shows a well-limited tissue structure, with a coarse oval shape, hyperechoic in relation to the homogeneous muscles, reminiscent of the thyroid parenchyma. It should be noted that the thyroid cavity was empty on ultrasound examination. Arrows 1 and 2 allow the estimation of the volume of the ectopic thyroid. It measures 21 × 9 mm.

The left arm measures 61 cm, the right arm measures 62 cm, and the right lower limb is 83 cm longer than the left by 3 cm, with a scoliotic attitude with flat feet. Based on this clinical assessment, the Netchine-Harbison​​​​​​​ score was 6/6 [1]. The biological workup showed profound hypothyroidism with TSH of 100 mUi/L, LT4 of 0.45 ng/mL (normal range: 0.7-1.48 ng/mL​​​​​​​), and anti-thyroid peroxidase​​​​​​​ (TPO) antibodies of 0.30 IU/mL < 5.61. An old thyroid checkup was normal according to the family, but we had no documents. Ultrasound images passing the upper limit of the hyoid bone showed a well-limited, roughly oval, homogeneous tissue structure measuring 21 ×​​​​​​​ 9 mm, reminiscent of thyroid parenchyma, and the thyroid compartment was empty on ultrasound exploration as can be seen in Figure 3.

Radiological exploration showed coxal bone irregularity bilaterally with no other renal, cardiac, or genital malformations. The bone age was estimated at 14 years with a two-year lag. The patient was put on L-thyroxine thyroid axis replacement and underwent TSH monitoring along with a genetic study.

## Discussion

Silver-Russell syndrome (SRS) was first described in 1953 [2]. It combines severe intrauterine growth retardation, postnatal weight-bearing delay, a particular facial dysmorphia, and limb asymmetry [3]. Our patient presents a height and weight delay with dysmorphic syndrome and body asymmetry, which was also reported in the literature [4,5]. In the study of Price et al. [6], 34% were asymmetrical, with a limb length discrepancy greater than 0.5 cm. Limb circumference was also affected in all of these cases. All asymmetrical subjects had a classical facial appearance. The lower limb was involved in all cases. Ten patients also had upper limb asymmetry, and in these, truncal and facial asymmetry was more often noticeable. The maximum leg length difference was 2.5 cm, whereas in our patient, the difference in the lower limbs was 3 cm, which was not previously reported in the literature. In another study [7], the authors showed that all patients with body asymmetry had scoliosis, which was also noted in our patient. Other manifestations were reported in the series [8,9], such as syndactyly of the second and third toes, clinodactyly, anorexia, and migraine, which were not found in our patient.

The clinical diagnosis of SRS is suspected when four out of six criteria of the Netchine-Harbison score [1] are present and molecular confirmation testing is warranted. It can stratify patients with SRS into subgroups, which can lead to more tailored management. However, molecular investigations come back negative in a notable proportion of patients with characteristic clinical features of SRS [7]. In our case study, we confirm the diagnosis of Silver-Russell syndrome based on the clinical description, and a genetic study is underway.

The mutations responsible for SRS are molecular abnormalities of the genes coding for fetal growth factors [10,11], by loss of methylation of the 11p15 chromosomal region 5 in 30%-60%, maternal uniparental disomy of chromosome 7 in 5%-10%, and chromosomal rearrangements involving imprinting center 1 (IC1) [11]. Other genetic abnormalities have been described, such as chromosome 15 deletion or chromosome 19 translocation. Most cases of Silver-Russell syndrome are sporadic [12,13]. The heterogeneity of the molecular abnormalities is at the origin of several phenotypes [14]. SRS is associated with several congenital malformations: genital, cardiac, gastric, and renal anomalies. However, thyroid malformation has never been reported [15]. Sublingual thyroid ectopy, which is present in our patient, is a rare condition related to a failure of migration of the thyroid gland during embryonic development [16]. The lingual thyroid is the most frequent form [16]. Besides the growth issues, neurodevelopment is of great concern to parents. Evidence shows that children with this condition are at increased risk for developmental delay (both motor and cognitive) and learning disabilities, which was indeed noted in our patient.

The treatment of SRS is symptomatic and multidisciplinary. The administration of growth hormone and surgical treatment by progressive bone lengthening of the femur or tibia by the internal system for functional and aesthetic purposes, indicated only in cases of inequalities of the lower limbs at the end of growth and in adulthood, as comorbidities must also be treated [16]. For thyroid dysgenesis, the treatment is substitutive in the long term. The prognosis is related to malignant degeneration, hence the interest in long-term clinical and biological monitoring [16]. Genetic counseling depends on the underlying molecular mechanism. The risk of recurrence is very low in the case of parental unidisomy of chromosomes [7]. This risk is higher in the case of a mutation of the imprinting center that is transmitted in a Mendelian fashion.

## Conclusions

Silver-Russell syndrome is a genetic disease linked to a parental imprinting anomaly. Its diagnosis is essentially clinical. Associated malformations are often described in SRS, hence the interest in a morphological assessment to optimize their management. Genetic counseling depends on the underlying molecular mechanism. Diagnostic difficulties in adulthood should make physicians and healthcare personnel aware of the need to take measurements, especially for newborns, in order to intervene early and improve medical, cognitive, and psychosocial management in childhood.
